# Development of a Core Outcome Set for Studies on Obesity in Pregnant Patients (COSSOPP): a study protocol

**DOI:** 10.1186/s13063-018-3029-1

**Published:** 2018-11-27

**Authors:** Rachel Dadouch Enzel, Mina Faheim, Clara Juando-Prats, Janet Parsons, Rohan D’Souza

**Affiliations:** 1 Institute of Medical Science, University of Toronto7, Toronto, Canada; 2 MD Program, University of Toronto, Toronto, Canada; 3 Applied Health Research Centre, Li Ka Shing Knowledge Institute, St. Michael’s Hospital, Toronto, Canada; 4 Department of Physical Therapy, University of Toronto, Toronto, Canada; 5 Division of Maternal-Fetal Medicine, Department of Obstetrics & Gynaecology, Mount Sinai Hospital, University of Toronto, 3-908 – 700 University Avenue, Toronto, ON M5G 1Z5 Canada

**Keywords:** Core outcome set, Obesity, Pregnancy, Childbirth, Stakeholder, Patient-reported outcomes, Outcome reporting, Qualitative research, Consensus, Delphi

## Abstract

**Background:**

Maternal obesity is a risk factor for adverse maternal, fetal, and neonatal events. Numerous clinical trials are currently exploring the effectiveness of antenatal and peripartum interventions in improving pregnancy outcomes that can in future inform clinical practice. However, the heterogeneity in outcome reporting limits our ability to compare outcomes across studies, and there is a lack of stakeholder representation in outcome choice. A pragmatic solution to this problem is the development of a core outcome set (COS) that defines the minimum criteria for outcome reporting in clinical trials undertaken in this population, arrived at by the involvement of relevant stakeholders.

**Methods:**

The development of a COS for studies on obesity in pregnant patients (COSSOPP) will comprise five steps. First, a systematic review of published literature will identify the long list of outcomes, their definitions and measurements if applicable, and outcome reporting quality. This will be followed by a meta-synthesis of qualitative studies with patients, and qualitative interviews in Toronto with patients, clinicians, researchers, hospital administrators, and policy-makers, to identify novel outcomes that were not obtained through systematic review. Third, the long list of outcomes will be narrowed down through online Delphi surveys involving an international group of patients and relevant stakeholders. This will be followed by a face-to-face consensus meeting with representatives of all stakeholder groups to arrive at a consensus on the final COS. Finally, in order to determine how the identified core outcomes should be measured, we will conduct another literature review and Delphi process.

**Discussion:**

COSSOPP will engage patients, clinicians, researchers, and other relevant stakeholders in determining the core set of outcomes that should be reported and measured in order to harmonize outcome reporting in studies evaluating the effectiveness of antepartum and peripartum interventions in obese pregnant women. This protocol provides a detailed overview of the steps involved in the development of a COS, to guide researchers in developing COS within their areas of specialization.

**COMET Core Outcome Set Registration:**

http://www.comet-initiative.org/studies/details/939.

**Electronic supplementary material:**

The online version of this article (10.1186/s13063-018-3029-1) contains supplementary material, which is available to authorized users.

## Background

An estimated 38.3% of women in the USA between the ages of 20–39 years have a body mass index (BMI) ≥ 30 [1], many of whom will plan a pregnancy. Not only do these women have higher incidences of pre-existing conditions such as diabetes mellitus, chronic hypertension, cardiovascular disease, and mental illness [2, 3], but even in their absence, are at a higher risk of developing pregnancy- specific complications during the antepartum (gestational diabetes mellitus, gestational hypertension, preeclampsia, fetal macrosomia, intrauterine growth restriction, congenital anomalies, and stillbirths [4–6]), intrapartum (labor dystocia, and failed labor induction or augmentation [7, 8] resulting in higher rates of cesarean and operative vaginal deliveries and birth trauma [9]), and postpartum (wound infections, hemorrhage, thromboembolism, depression, lactation failure, and prolonged hospitalization [5]) periods.

Since pre-conception weight loss interventions (apart from bariatric surgery) have demonstrated limited benefit in improving pregnancy outcomes [10, 11], and most pregnancies are unplanned, the focus of research is geared to reducing complications in mother and baby via antepartum and peripartum (a term used to describe intrapartum and postpartum) interventions [12]. We have identified 30 clinical trials (CTs) and 25 trial registrations and/or protocols involving obese pregnant women and these numbers are only expected to increase. However, systematic reviews and meta-analyses of CTs looking at these interventions repeatedly highlight heterogeneity in outcomes reported between studies, causing difficulty with data aggregation and synthesis [13, 14]. This, along with the paucity of patient-reported outcomes undermines the external validity of published studies in clinical practice [15]. International initiatives, such as Core Outcome Measurement in Effectiveness Trials (COMET) and Core Outcomes in Women’s and Newborn Health (CROWN) have addressed the need to develop “core outcome sets” (COS) aimed at standardizing the reporting and measuring of health outcomes in research [15, 16].

A COS is a standardized, minimum set of outcomes, agreed upon by patients and those involved in their care (stakeholders) that should be reported and measured in all clinical trials of a specific condition [15]. The importance of COS has been recognized and endorsed by researchers and journal editors worldwide, and 49 COS protocols and four completed COS have been identified in women’s and newborn’s health alone [17]. Although there are published recommendations on the framework and standards for COS development [15, 18], each COS needs to be designed based on specific attributes of the scope, setting, condition, population, and intervention(s) [18] in question. We present a protocol for the development of a COS to be used in future trials exploring interventions to improve outcomes for obese pregnant women.

## Methods

The development of this protocol was guided by the COMET Handbook [15] and the COS-STAndards for Development (COS-STAD), with adaptations specific to the scope of this project [18]: the Core Outcome Set for Studies on Obesity in Pregnant Patients (COSSOPP).

The outcomes determined throughout development, and thus the resulting core outcomes, will be categorized according to their applicability in antepartum, peripartum or both antepartum and peripartum interventions, as some outcomes (e.g. development of gestational diabetes mellitus) are relevant only to the antepartum period and other outcomes (e.g. wound complications due to cesarean delivery) are relevant merely to the peripartum period. Additionally, a subset of outcomes applicable to solely anesthetic interventions may result in a separate category as well.

**Fig. 1:**
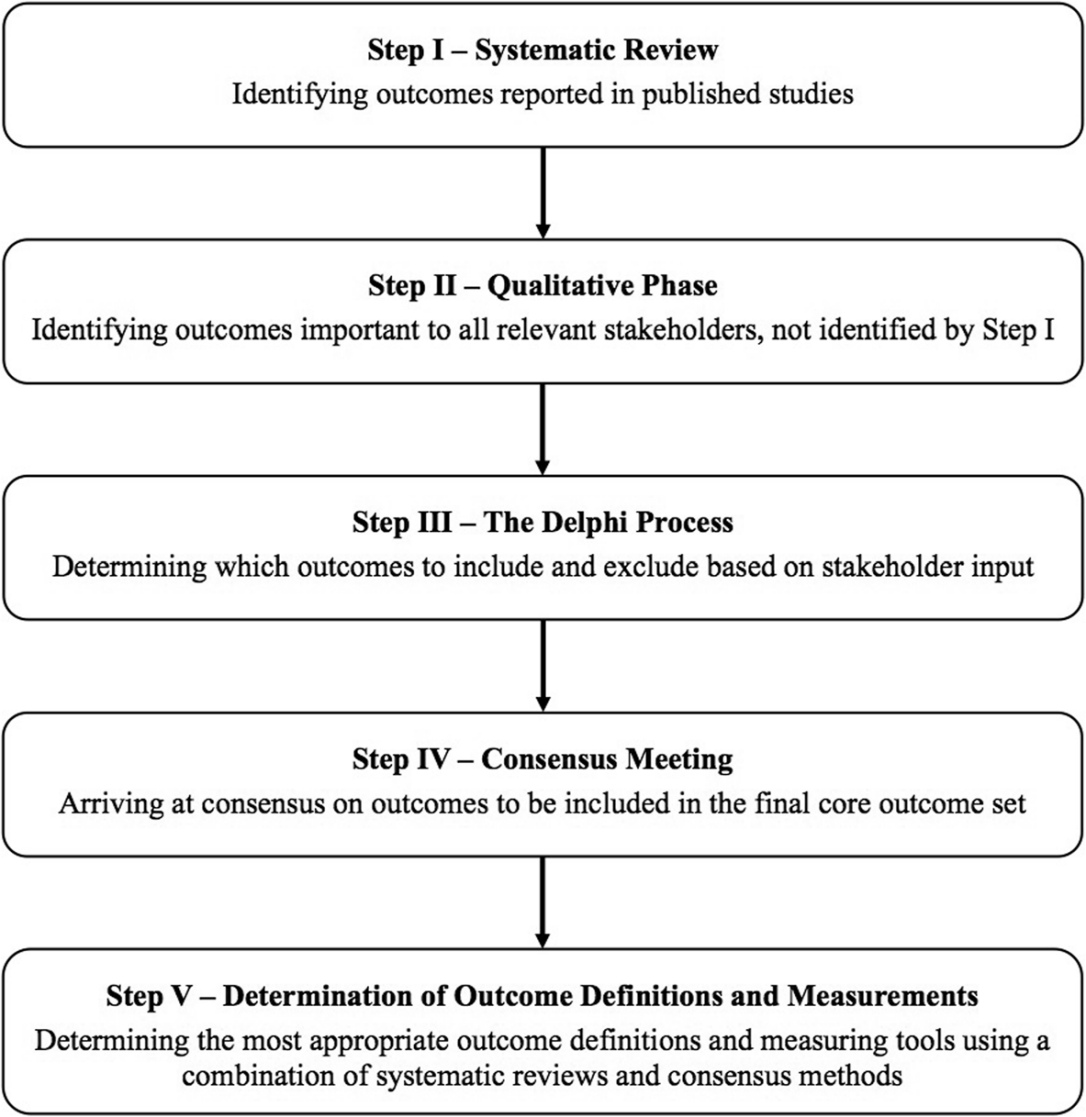
Five steps in the development of a core outcome set

The steering committee has registered COSSOPP on the COMET website [19] and will oversee the five steps involved in its development (Fig. 1):

### Step I: systematic review

All reported primary and secondary outcomes in studies involving interventions and exposures in obese pregnant women will be identified through a systematic review of published studies. The aim of this step is to generate a preliminary list of outcomes reported (and therefore considered important) by researchers. The systematic review will be conducted and reported in accordance with the Preferred Reporting Items for Systematic Reviews and Meta-Analyses (PRISMA) guidelines and the protocol is available on PROSPERO, the international prospective register of systematic reviews (CRD42017080279) [20]. The primary research question is “What maternal and fetal/neonatal outcomes have been reported in studies involving antepartum or peripartum interventions/exposures in pregnant women with body mass index (BMI) ≥30?” and the secondary question is “How have these reported outcomes been defined and/or measured?” Features unique to a COS systematic review have been highlighted below.

#### Study selection

We will employ the OvidSP platform with MeSH terms and keywords related to obesity in pregnancy, and include three bibliographic databases - Medline, Embase, and ClinicalTrials.gov. As COS seek to standardize outcome reporting in CTs, we will include all CTs and trial registrations in our systematic review. In addition, to ensure that outcomes reported in observational studies are also represented, we will include systematic reviews of all study types.

#### Data extraction and synthesis

**Fig. 2:**
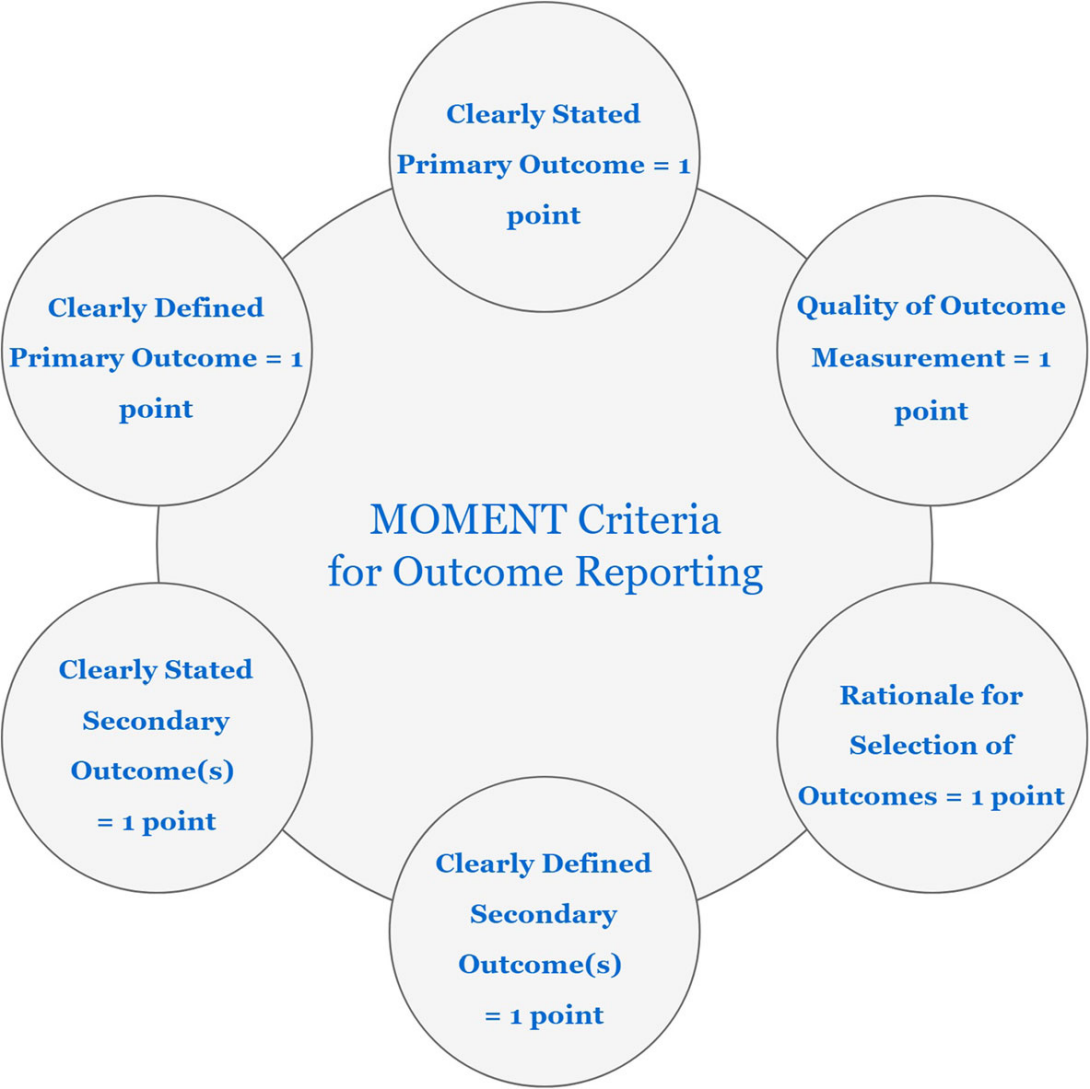
The Management of Otitis Media with Effusion in Children with Cleft Palate (MOMENT) criteria toassess the quality of outcome reporting

Data on year of publication, study type, country, number of participants, and broad (antepartum versus peripartum) intervention category will be extracted from included studies. All reported primary and secondary outcomes, including composite outcomes and their definitions and measurement tools will be listed. It is vital for this systematic review that all reported outcomes are identified and assigned equal importance while proceeding to the next stage. This includes outcomes reported in studies that would otherwise qualify as “low-quality” or “at high risk-of-bias”, based on assessment of the study’s methodological quality. In order to avoid this, and as our primary intention is the assessment of outcome reporting, no assessment of the study’s methodological quality will be performed. Instead, we will use the criteria established by The Management of Otitis Media with Effusion in Children with Cleft Palate (MOMENT) [21] group to assess outcome reporting quality. Studies will receive a score from 1 to 6, with scores > 4 representing high outcome-reporting quality (Fig. 2). Although these criteria have not been validated and are not considered an essential part of systematic reviews for COS development, they might suggest correlation or lack thereof between reporting quality and the nature of outcomes reported.

#### Analysis and presentation of results

We will document the proportion of studies reporting each primary and secondary outcome, the proportion that do not report a primary outcome at all or report it in a non-reproducible way and the components and definitions of each primary, secondary, and composite outcome. Subgroup analyses will be performed based on (1) the timing of intervention (antepartum versus peripartum) and (2) study type, i.e. CTs, trial registrations, secondary analyses of CTs, or systematic review. Identified outcomes will be grouped into broader domains according to a recently developed taxonomy for outcomes [22].

### Step II: qualitative phase

The qualitative phase in COS development aims to identify outcomes that relevant stakeholders deem important, particularly those that were not determined in step I. The outcomes resulting from the qualitative phase will inform subsequent Delphi methodology [23]. Step II will consist of a meta-synthesis of qualitative literature followed by prospective qualitative interviews.

Review and synthesis of qualitative studies of obese pregnant women will be conducted in order to determine relevant themes and emerging patient-reported outcomes [23, 24], using the published taxonomy of outcomes in medical research as a guide [22]. This review has also been registered on PROSPERO [25]. As a preliminary search of the literature identified no studies primarily aimed at determining outcomes considered important by obese pregnant women, care providers and other stakeholders involved in their care specifically for the purposes of informing a COS, we will conduct qualitative interviews involving all relevant groups of stakeholders [15].

We identified four groups of stakeholders as vital to the development of a COS involving obese pregnant women: patients and partners or family members; clinicians (including obstetricians, family physicians, nurses, midwives, anesthesiologists, ultrasonographers, dieticians, and social workers); researchers with expertise in obstetric outcomes/obstetric health services research; and others (mainly hospital administrators and policy makers). Bringing diverse stakeholders together to try and reach a consensus is seen to be the future of collaborative and influential research [15]. We will therefore invite these four groups of stakeholders to participate in focus groups or individual in-depth interviews using interpretive description [26], to identify outcomes that they consider important for the health management of this population.

#### Sampling

Patients and/or their family members or spouses (group A) will be recruited from our center and interviewed separately from other stakeholders (group B) to avoid power imbalances affecting the focus group dynamics [27], and to separate different stakeholders’ perspectives. Group-B focus groups will consist of an assortment of colleagues at our center, Mount Sinai Hospital, with various roles that involve experience caring for pregnant women with obesity. In group A, focus groups of nulliparous women will be conducted separately from those of multiparous women. The data in focus groups rely on the natural, emergent dialogue between participants, therefore purposively sampling the patient stakeholder group into sub-categories according to parity provides commonality within the group that may help with dialogue initiation and flow [28].

#### Data collection

As stated, focus groups and individual interviews will be the two methods of data collection. Focus groups yield rich data in a time-efficient way and utilize participant dialogue as a catalyst for the sharing of experiences, preferences, and values between participants, evoking the emergence of valued outcomes [28]. However, recruitment success may influence the ability to comprise a full focus group, leading to the conducting of individual interviews specifically for patients [27]. Factors that can limit recruitment success for a pregnant patient population at our center include gestational age preventing attendance close to labor, travel distance from place of residence, child care, and comfort level in a group setting. In terms of the latter phenomenon, if both focus group and individual interviews are conducted, they will complement one another as data that would not emerge in a group setting could surface in a one-on-one dynamic. As well, individual interviews can enhance richness to the findings of the focus groups, providing an opportune platform to elaborate on certain outcomes [27, 29].

Variation within participant groups will be accounted for in data analysis by linking features such as BMI, gestational age, co-morbidities and demographic information (age, occupation, education, ethnicity) to the outcomes determined, for the patient stakeholders. We will collect this information the day of the focus group or individual interview subsequent to the consent process in an optional form, and the more personal medical characteristics (e.g. BMI) from patient charts after the qualitative interview, which is after having taken patients’ consent as well. We expect a diverse group of participants in regard to co-morbidities and demographics, as Mount Sinai Hospital is a tertiary care center in a diverse urban setting. Age, ethnicity and occupation in the remaining stakeholder groups will be collected from the professional stakeholders in a similar fashion, subsequent to consent prior to the start of the focus group or individual interview session. All transcripts will be anonymized and data will be saved in password-protected folders on a secure hospital hard-drive.

#### Interview guide and structure

Focus groups will comprise 6–10 participants, who will be asked to provide outcomes they consider relevant to consider and measure in this research area (without prompting) and their rationale (see Additional files 1 and 2), until no new outcomes emerge. We will then ask participants to comment on the domains of outcomes identified in the systematic reviews, using comprehensible language when appropriate. Focus groups will be recorded, later transcribed and analyzed and will continue until no new outcomes are identified in two successive sessions, referred to as “saturation” [27]. For the in-depth individual interviews, via telephone or in person depending on the patient’s preference, the interview guide will be similar to that of the focus groups (see Additional file 3). In focus groups and individual interviews, one researcher will conduct the audio-recorded interview, and a second researcher will take field notes [29].

#### Data analysis

We will analyze data throughout the data collection period according to interpretive description, which is an inductive, non-categorical qualitative approach [26] using thematic analysis. First, general notes will be taken from the transcripts and thereafter a second read through will be undertaken in more detail, using line-by-line color coding to group related concepts into themes or domains. Outcomes will then be identified from each theme [27]. For example, “wound complication concerns” may emerge as a theme, in which surgical site infection or bloody discharge may surface as outcomes. The researcher who conducted interviews and focus groups will analyze all data, while a second researcher will analyze a subset of transcripts and complete a 10% verification check of analysis. Major discrepancies will be resolved by discussion with a third researcher [27]. Merging outcomes acquired in this step with those obtained through step I, will constitute the long-list of outcomes to inform the next step [23].

### Step III: the Delphi process

The long-list of outcomes will be condensed using the Delphi method to achieve convergence of opinion in an iterative and sequential manner [17, 30].

#### Developing the survey

We will initially stratify outcomes under three headings – “all studies”, “antepartum only” and “peripartum only”, and further into clinical domains for clarity and consistency [15]. Collaborating with certified linguists, we will develop lay-language descriptions that will appear alongside the identified outcomes where necessary. The survey will be piloted on a representative sample of stakeholders through face-to-face interviews to obtain input on the use of open-ended questions [30], structuring of survey items [15, 31], survey length [15], and the adequacy of lay-language summaries.

#### Delphi panels

The approved survey will be distributed to Delphi panel members using DelphiManager™ software, to ensure anonymity, feasibility, reproducibility, and cost effectiveness [32] while facilitating global representation. Members from each stakeholder group will be invited to participate. Participants will be identified and recruited through various channels: (1) patients and their partners or family members, through obesity-in-pregnancy clinics, mother-baby blogs and patient-advocacy groups; (2) researchers through author lists of papers included in step 1; (3) clinicians through members of national and international societies of obstetrics, gynecology and obesity and participant lists published by international conferences and steering committee members’ contacts; and (4) other stakeholders through author lists of published guidelines and known administrators of healthcare units. Attempts will be made to ensure global representation.

#### Group size

To maximize the external validity of our findings, adequate representation from each of the four identified stakeholder groups will be sought. However, there are currently no definitive, evidence-based recommendations about the ideal group size for a Delphi survey, and decisions on the number in each stakeholder group are expected to be influenced by the scope of the COS, existing knowledge, and practical feasibility considerations [15]. As such, we will aim to recruit a minimum of 25 individuals from each stakeholder group to ensure sufficiently diverse representation. However, the emphasis will be on recruiting as many as possible of their patients and healthcare providers involved in their care.

#### Obtaining consent and demographic details

Those who demonstrate interest in participating will receive an email with a link to the Delphi survey, clicking on which will provide information on the survey’s purpose and the importance of participating in both rounds and instructions, which may be used as a reference throughout the process. Once the consent form is signed electronically, participants will be required to fill in a brief demographic questionnaire to indicate with which of the four stakeholder groups they primarily identify, their specific role (e.g. current or former patient, research nurse, dietician, etc.), age, country of residence, sex, and experience with COS development. Participants will have the option of declining to answer all but the first question pertaining to the primary stakeholder group, since this variable is essential for generating feedback between rounds, and to ensure that the final COS includes outcomes considered important by each stakeholder group.

#### Delphi rounds

There will be two rounds of surveys. The first round will ask participants to score each outcome on a 9-point Likert scale based on the degree of importance as recommended by the Grading of Recommendations Assessment, Development and Evaluation (GRADE) working group [33]. Scores of 1–3 will be regarded as “not essential”; 4–6, “important but not critical”; and 7–9, “critically important for inclusion”. Those lacking experience or expertise to evaluate specific outcomes will be permitted to select “unable to score”. Participants will also have an opportunity to add additional outcomes in an open field at the end of the survey, accompanied by an assigned score on the Likert scale. Results of the first round will be analyzed and organized by stakeholder group, and the mean score assigned by all participants and those of each stakeholder group for each outcome will be graphically presented and made available to participants in the second round along with the score they originally assigned. Participants can choose to alter their responses based on this new information, or retain the original score. All outcomes from the first round will be carried forward to the second round regardless of the score assigned. Prior to submission of the second-round survey, participants will be informed of the next step in COS development, a face-to-face consensus group meeting and after providing a brief explanation, asked if they would be interested in participating.

#### Response rates and attrition

Attrition rates in published COS within women’s and newborn’s health, have varied from 21 to 48% [17]. To maximize response rates and minimize attrition bias, several strategies will be used including a 6-week window to complete each survey round, bi-weekly reminder e-mails, and clearly outlined expectations in the participant information sheet. Should these measures be insufficient to secure a response rate of 80%, which has been deemed acceptable based on published recommendations [15], additional strategies will be considered including extending survey deadline(s), telephone calls, and personalized reminders. Specifically, if the response is inadequate, the steering group will convene to determine whether keeping the survey window open for a longer period of time is likely to improve the response rate.

#### Defining and assessing consensus

For outcomes to be considered in the final COS, they must meet pre-defined criteria for consensus within each group of stakeholders: “consensus in” - an outcome deemed “critically important” by ≥ 70% and “not essential” by ≤ 15%; “consensus out” - an outcome deemed “critically important” by ≤ 15% of each stakeholder group and “not essential” by ≥ 70% and “no consensus” - any other outcome.

### Step IV: consensus meeting

Participants from the wider Delphi cohort that expressed interest in participating will be invited, and two to five members of each stakeholder group will be randomly selected to ensure equal representation from stakeholder groups whilst acknowledging logistical barriers that will prevent many participants from attending in person. Although there is format for a consensus meeting in the context of COS development, it is generally viewed as a valuable means of facilitating interactive debate between stakeholders and resolving inclusion/exclusion conflicts [15]. Our half-day meeting will take the form of a guided open discussion led by members of the steering committee. Its purpose is twofold: to achieve consensus on items in the no-consensus category, and for a vote on the final COS. Stakeholders will first share their perspectives about items in the no-consensus category, followed by an electronic anonymous vote, which will employ the same scoring system and consensus thresholds as in step III. For each of the three study categories - “all studies”, “antepartum” and “peripartum” - these new items and those already meeting criteria for consensus-in will be subjected to a final vote to determine the final COS. Although COS in obstetrics and gynecology have included between 11 and 48 outcomes [17], since our aim is to define a minimum number of outcomes to be included in all prospective studies and to maximize uptake by researchers, we will aim to restrict this to the smallest possible number, considering the modular nature of the COS that might include different outcomes for studies in the antepartum and peripartum periods. We will, however, ensure that no outcomes considered critically important by any of the stakeholder groups are eliminated, merely to arrive at this small number.

### Step V: determination of outcome definitions and measurements

We will use the consensus-based standards for the selection of health measurement instruments (COSMIN) to evaluate each identified outcome measuring tool based on four criteria: validity, reliability, responsiveness, and interpretability [34]. Measurement instruments and definitions will be identified through a separate systematic review in addition to studies included in step 1 [35]. Another Delphi process employing similar consensus methods, and involving representatives of the above stakeholder groups will determine the most appropriate outcome measuring tool(s) for each identified core outcome.

### Knowledge translation

COSSOPP findings will be presented at international obstetrics and obesity meetings, published in an open-access journal and archived in the COMET and CROWN databases.

## Discussion

We present a protocol for the development of a COS comprising the minimum set of outcomes to be included in future studies involving obese pregnant women. Once published, researchers studying the effectiveness of antepartum and peripartum exposures and interventions in this patient population will have an empiric basis for inclusion of outcomes, based on input from patients and other stakeholders involved in their care. This would decrease reliance on surrogate outcomes and prevent outcome reporting bias. Furthermore, authors of systematic reviews will be better equipped to meta-analyze data to guide clinical decision-making. Although our COS is designed for studies on obese pregnant women, we hope that the principles we have outlined encourage development of similar COS in other areas of obesity research where standardization of outcome reporting remains a concern.

## Additional files

Additional file 1: Focus Group Interview Guide – Step-II COSSOPP: Session with Patients. An interview guide with transitions, questions and prompts to conduct a focus group session with patients. (DOCX 22 kb)

Additional file 2: Focus Group Interview Guide – Step-II COSSOPP: Session with Professionals. An interview guide with transitions, questions and prompts to conduct a focus group session with health care professionals. (DOCX 24 kb)

Additional file 3: One-on-One Interview Guide – Step-II COSSOPP: Patients An interview guide with transitions, questions and prompts to conduct a one-on-one interview with patients either via telephone or in person. (DOCX 22 kb)

## Abbreviations

BMI: Body mass index
CT: Clinical trial
COMET: Core Outcome Measurement in Effectiveness Trials
COS: Core outcome set
COSMIN: Consensus-based standards for the selection of health measurement instruments
COSSOPP: Core outcome set for studies on obesity in pregnant patients
CROWN: Core Outcomes in Women’s and Newborn Health
