# Supplementary material for: Development of a Core Outcome Set for Studies on Obesity in Pregnant Patients (COSSOPP): a study protocol
Source: Trials. 2018 Nov 27;19:655. doi: 10.1186/s13063-018-3029-1 (PMC6258169; doi:10.1186/s13063-018-3029-1)
Supplement: Supplementary file 1 — Focus Group Interview Guide – Step-II COSSOPP: Session with Patients. An interview guide with transitions, questions and prompts to conduct a focus group session with patients. (DOCX 22 kb) [file 13063_2018_3029_MOESM1_ESM.docx]

**Focus Group Interview Guide – Step-II COSSOPP**

**Session with Patients**

**July – September 2018**

Preamble:

*Thank you everyone for joining this focus group session today. We truly appreciate your time. You are here today to participate in one step that is part of a larger study. We are creating a research “tool” called a Core Outcome Set (COS), which is becoming more popular in medical research because it will fix many of the problems that exist in how well research is conducted. One of the main problems is the lack of everyone’s perspective in research, including the perspective of patients. A COS in our field involves finding out everyone’s perspective when it comes to pregnancy, labour, delivery and postpartum, and translating what we find out from you into research and medicine. We are also interviewing doctors, researchers, nurses, and other professionals who are involved in your care. We are coming up with a long list of everyone’s considerations, and after we complete this step, we will conduct surveys to narrow down this long list into the core set of things to measure in research going forward. Therefore, it is important that we discuss with you what you feel must be considered when it comes to you or your baby, so that these opinions can make it to the next stage.*

*We will start by facilitating discussion on what you think must be accounted for in the pursuit of your optimal health and well-being, and we then will share some results from our review of the research literature, so you can comment on and evaluate on what researchers are currently reporting.*

*Your opinions and perspectives can be in any category, from mental health, physical health, social considerations that relate to your partner, family or friends, and more. Questions and topics we present serve as a guide only, and are not meant to limit your perspective. Please raise anything that we have not specifically asked about. Challenges, successes and experiences of all kinds, both for you and your baby, may be relevant and we welcome you to share.*

*This session will be recorded for analysis and kept until study publication when it will be destroyed. If at any time you would like to avoid answering a question, take a break or leave the focus group, please do so. Results obtained from the focus group will have no names or identifiers, and will be kept strictly confidential. Once transcribed, the data will be analyzed and used as a starting point for the next steps of the study. Our study personnel have received REB approval from Mount Sinai Hospital.*

*We want to hear your perspective and use it to guide future research and care for women in the Special Pregnancy Program. We hope today’s session will be a good opportunity to do so. Thank you once again for agreeing to participate. The focus group discussion will last for around 60-75 minutes. Does anyone have any questions? If not, let’s begin.*

Questions

1. Describe some experiences during pregnancy and how your life has been affected? Are there some experiences that have significantly changed the way you live in terms of family, friends or as an individual?

*Potential probe*

- What has managing your pregnancy and upcoming childbirth been like?

*Assume participants will provide some experiences in pregnancy.*

1. As you progress through your pregnancy, are there more considerations, concerns or other experiences that arise for you? Which of these have been the most important that you think about day to day?

   *Assume participants will provide some highlighted experiences.*
2. When it comes to your baby specifically, what do you think most about? What aspects of their health do you take into consideration?

   *Assume participants will share outcomes related to baby.*
3. Overall in terms of yours or your baby’s health, what matters most to you?

   *Assume participants will provide some highlights of their main priorities.*
4. Throughout the pregnancy have there been serious challenges that were difficult to go through? Was there a diagnosis or piece of news that was difficult to receive, or that you didn’t expect to receive? (Please share these if you’re comfortable).

   *Follow up:*What did your health care provider say when delivering this information to you?

   *Assume participants will share some co-morbidities or other challenges.*
5. What are the main pieces of advice you would share with a friend going through pregnancy and labour? We’re interested in hearing about any trimester, and any aspect of your treatment, care, health or well-being.

*Assume participants will share major experiences and considerations that are at the forefront of their minds.*

1. Is there anything that you have been thinking about related to your pregnancy and having a child, that you feel does not get addressed? What are some of these concerns, issues, or experiences that have been dismissed in your pathway of care?

   *Assume either no input, or some concerns that have not been addressed.*
2. Has there been any considerations or pieces of advice that your health care provider has shared with you that you don’t agree with, or that you feel wasn’t really important?

   *Follow up or prompts depending on discussion:
   -* Which considerations do you think may have been over-emphasized?
   - What were your thoughts or feelings through genetic tests, ultrasounds, etc.?

   *Assume participants will share concerns of HCPs that they don’t have.*
3. Before we move on to the findings of our review of the literature, are there any other aspects of your health and care that we did not touch upon that you want to share?

   *Assume either silence, nods no or if yes, an answer to this question.*
4. In the literature, researchers typically have reported on: diet and exercise measures, wound complications, blood pressure, GDM, pain after caesarean deliveries, gestational weight gain, postpartum weight retention, preeclampsia, initiation of breastfeeding, length of hospital stay, and more. Upon reflecting on one, any or all of these, what are your thoughts?

   - Have you discussed any or most of these with your health care provider?

   - Which ones, and how often?

   *Prompt:*- Is there anything missing from this list that you would like to add?
5. To sum up after having these discussions and hearing several perspectives, could you provide us with two or three main considerations that you think are essential to consider, and should be measured in research? These can relate to you or your baby, during pregnancy and beyond.

   *Expect valued outcomes, hopefully from all participants.*
6. Is there anything else anyone would like to add?

   *Either silence, or some minor additions.*
7. Thank you all for attending and participating. Please fill out the feedback form on your way out, which will ask about your possible participation in an online survey, a few months from now. Let any of us know if you have any questions.
