# Supplementary material for: Development of a Core Outcome Set for Studies on Obesity in Pregnant Patients (COSSOPP): a study protocol
Source: Trials. 2018 Nov 27;19:655. doi: 10.1186/s13063-018-3029-1 (PMC6258169; doi:10.1186/s13063-018-3029-1)
Supplement: Supplementary file 2 — Focus Group Interview Guide – Step-II COSSOPP: Session with Professionals. An interview guide with transitions, questions and prompts to conduct a focus group session with health care professionals. (DOCX 24 kb) [file 13063_2018_3029_MOESM2_ESM.docx]

**Focus Group Interview Guide – Step-II COSSOPP**

**Session with Professionals**

**July – September 2018**

Preamble:

*Thank you everyone for joining this focus group session today. We appreciate your time. As part of the several Core Outcome Sets (COS) being developed in high-risk obstetrics at Sinai, one of them is in obesity in pregnancy trials. A COS is the minimum set of outcomes that will be reported on in clinical trials of a designated area. This standardization ensures the same outcomes at minimum are reported in all trials, allowing for many benefits such as study comparability, minimized researcher bias and uniform outcome definitions. It also includes input from the relevant stakeholders so that the outcomes measured have fairly weighed all perspectives, including those of patients. In the development of this COS, engaging stakeholders first begins with gathering perspectives, in order to come up with a long list of outcomes. The many outcomes in this long list will later be administered into surveys and scored individually, to eventually arrive at the core set of outcomes.*

*To create the initial long list, we’re conducting interviews and focus groups with patients and those involved in their care. Your insight and perspectives are valuable to us, in terms of what you believe matters most when caring for women of a BMI>30 whilst pregnant, in labour, delivery, postpartum and beyond. Health outcomes can categorize within any domain, from psychosocial to physical considerations.*

*Questions and topics we present serve as a guide only, and are not intended to limit your perspective. Please raise anything with respect to these women and their treatment or care. Challenges, successes and experiences that are maternal, fetal or neonatal may be relevant, and we welcome you to share.*

*This session will be recorded for analysis and kept until study publication when it will be destroyed. If at any time you would like to avoid answering a question, take a break or leave the focus group, please do so. Results obtained from the focus group will have no names or identifiers, and will be kept strictly confidential. Once transcribed, the data will be analyzed and used as a starting point for the next steps of the study. Our study personnel have received REB approval from Mount Sinai Hospital.*

*We want to hear your perspective and utilize these varying outlooks to guide future research and care for these women, so we hope today’s session will be a good opportunity to do so. Thank you once again for agreeing to participate. The focus group discussion will last for around 60-90 minutes. Does anyone have any questions? If not, let’s begin.*

Questions

1. What are some of your experiences with women with obesity throughout pregnancy until the time that you care for them? These can apply to any trimester of pregnancy or childbirth.

*Potential probe*

- What has managing their pregnancy and childbirth been like?
- Based on experience, what are some considerations you typically have with these patients?

*Assume participants will provide some experiences they have with this population, to stimulate conversation on the topic in general.*

1. Compared to normal or overweight women, how do your roles differ with women of a BMI > 30 specifically?

   *Assume participants will provide some differences between caring for this group of women and other non-obese women.*
2. What are some experiences or circumstances that are typical of or unique to this group?

   *For diagnoses or specific outcomes that may emerge:*- Are there any discrepancies in the definition of this outcome? How do you define that diagnosis compared to others in the same or similar role to you?

   *Assume participants will share what they see or experience frequently with this population, as well as discrepancies in outcomes that may have emerged.*
3. Overall, what aspects of the mother’s or baby’s health matter most to you when working with, or caring for these women?

*Assume participants will provide some unique considerations for this group.*

1. What are your considerations when it comes to fetal, neonatal or infant health?

   *Assume outcomes emerge relating to fetal/neonatal health.*
2. What are the main pieces of advice you would share with a colleague who does not treat or care for this population as frequently as you do?

   *Potential prompt:*- What is your greatest challenge or success with this group of women that could be passed on to other professionals?

*Assume participants will provide the highlights or main considerations.*

1. Do you notice any gaps in care for these women? If so, what might be some of their concerns, health issues, or experiences that are dismissed in their care?

   *Assume either no input for this question, or some examples.*
2. What are some concerns or positive experiences that patients with obesity typically share with you?

   *Probe*
   This may include topics that are related to the partner or family, comfort, physical health, mental health, daily routine or quality of life, decision-making, etc.

*Assume people will share anecdotes and specific examples of patients’ concerns that were raised by patients, as told through the lens of the professional.*

1. Are there any differences between what you and the patient deem important?

   *Follow up questions:*- Describe situations in which these patients have exhibited non-compliance.
   - What might be the causes of any of these scenarios?

   *Expect outcomes that they deem important, that patients may disagree with.*
2. Upon reflecting on past experiences, what have been patients’ responses to complications or diagnoses that you discussed with them?

*Probe:*
- Patients’ responses can pertain to before or after the occurrence of a complication.

*Assume participants will share scenarios that reveal more on their relationships with this patient group.*

1. Which considerations do you have that you think other professionals over-emphasize, or conversely, need to be more emphasized when caring for these women?

   *Assume a discussion of outcomes, in terms of what should be valued more or less.*
2. What are the ten outcomes you value most when it comes to pregnant women with obesity, and should definitely be measured in this research area?

   *Follow up:*- Please elaborate your reasoning, especially if discrepancies arise between your top ten outcomes.

   *Expect most valued outcomes and subsequent discussion.*
3. Before moving on to the findings of our Systematic Review, are there any aspects of these women’s care that we did not discuss?

   *Assume either silence, nods no or if yes, an answer to this question.*
4. Outcomes that are reported in the literature include: diet and exercise measures, wound complication outcomes, blood pressure, GDM, postoperative pain, weight-related (GWG, PPWR, etc.), preeclampsia, initiation of breastfeeding, length of hospital stay.

   - Do you have any immediate opinions on any of these or this list in general?

   *Specifically choose ones that did not come up in session thus far:*
   - Why do you think this outcome *x* did not come up in conversation, or was not discussed at greater length?

   *Assume discussion on some outcomes from the systematic review results.*
5. For any of these outcomes (we can repeat shortly), are there pertinent details or definitions that you can offer? For example, with wound complications, what should be included to appropriately measure this outcome?

   *Assume elaboration on outcomes, and definitions or measurements.*
6. To sum up, after discussing and listening to various perspectives, could you provide us with two or three outcomes that you deem essential to consider?

   *Expect valued outcomes from participants.*
7. Is there anything else anyone would like to add?

   *Either silence, or some minor additions.*
8. Thank you all for attending and participating. Let any of us know if you have any questions.
