# Supplementary material for: Development of a Core Outcome Set for Studies on Obesity in Pregnant Patients (COSSOPP): a study protocol
Source: Trials. 2018 Nov 27;19:655. doi: 10.1186/s13063-018-3029-1 (PMC6258169; doi:10.1186/s13063-018-3029-1)
Supplement: Supplementary file 3 — One-on-One Interview Guide – Step-II COSSOPP: Patients An interview guide with transitions, questions and prompts to conduct a one-on-one interview with patients either via telephone or in person. (DOCX 22 kb) [file 13063_2018_3029_MOESM3_ESM.docx]

**One-on-One Interview Guide – Step-II COSSOPP**

**Patients**

**July – September 2018**

Preamble:

*Thank you for your willingness to talk to us today. We truly appreciate your time. This interview is one step that is part of a larger study. We are creating a research “tool” called a Core Outcome Set (COS), which is becoming more popular in medical research because it will fix many of the problems that exist in how well research is conducted. One of the main problems is the lack of everyone’s perspective in research, including the perspective of patients. A COS in our field involves finding out everyone’s perspective when it comes to pregnancy, labour, delivery and postpartum, and translating what we find out from you into research and medicine. We are also interviewing doctors, researchers, nurses, and other professionals who are involved in your care. We are coming up with a long list of everyone’s considerations, and after we complete this step, we will conduct surveys to narrow down this long list into the core set of things to measure in research going forward. Therefore, it is important that we discuss with you what you feel must be considered when it comes to you or your baby, so that these opinions can make it to the next stage.*

*We will start by facilitating discussion on what you think must be accounted for in the pursuit of your optimal health and well-being, and we then will share some results from our review of the research literature, so you can comment on and evaluate on what researchers are currently reporting.*

*Your opinions and perspectives can be in any category, from mental health, physical health, social considerations that relate to your partner, family or friends, and more. Questions and topics we present serve as a guide only, and are not meant to limit your perspective. Please raise anything that we have not specifically asked about. Challenges, successes and experiences of all kinds, both for you and your baby, may be relevant and we welcome you to share.*

*This interview will be recorded and observed for analysis and kept until study publication when it will be destroyed. If at any time you would like to avoid answering a question, take a break or do not feel comfortable, please tell me so that I can make sure you are accommodated. Results obtained from this interview will have no name or identifier, and will be kept strictly confidential. Once transcribed, the data will be analyzed and used as a starting point for the next steps of the study. Our study personnel have received ethics approval from Mount Sinai Hospital.*

*We want to hear your perspective and use it to guide future research and care for women in the Special Pregnancy Program. We hope today’s interview will be a good opportunity to do so. Thank you once again for agreeing to participate. This interview will last for up to 60 minutes. Do you have any questions? If not, let’s begin.*

Questions

1. Can you tell us about what your environment is like during your pregnancy?

   For example, if you live with anyone, have any other children or older adults you take care of, your day-to-day responsibilities, etc.?

   *Assume participant will provide share what her support system, responsibilities, etc. are at this time.*
2. Describe some experiences during pregnancy and how your life has been affected? Are there some experiences that have changed the way you live in terms of family, friends or as an individual?

*Probes*

- What has managing your pregnancy and upcoming childbirth been like?

- What is your support system like?

*Assume participant will provide some experiences in pregnancy.*

1. What are the most significant changes you’ve had in pregnancy until now?

   *Gathers changes and considerations if former questions did not successfully do so.*
2. As you progress through your pregnancy, are there more considerations, concerns or other experiences that arise for you? Which of these have been the most important that you think about day to day?  *Follow up question:*What are the main pieces of advice you would share with a friend going through pregnancy and labour? We’re interested in hearing about any trimester, and any aspect of your treatment, care, health or well-being.

*Assume participant will share major experiences and considerations that are at the forefront of their minds.*

1. When it comes to your baby specifically, what do you think most about? What aspects of their health do you take into consideration?

   *Assume participant will share outcomes related to baby.*
2. Overall in terms of yours or your baby’s health, what matters most to you?

   *Assume participant will provide some highlights of their main priorities.*
3. A) Throughout the pregnancy have there been life challenges that were even more difficult to go through because you are/were pregnant? These can relate to your support from family, friends and others, financial or work-related challenges, etc.?

B) How about medical-related challenges? Was there a diagnosis or piece of news that was difficult to receive, or that you didn’t expect to receive?

*Follow up:*What did your health care provider say when delivering this information to you?

*Assume participant will share life +medical challenges, in terms of pregnancy.*

1. Is there anything that you have been thinking about that you feel does not get addressed? *If yes:* What are the issues or experiences that were dismissed?

   *Assume either no input, or some concerns that have not been addressed.*
2. Have there been considerations or pieces of advice that your health care provider shared with you that you don’t agree with, or that you feel wasn’t really important?

   *Probes
   -* Which considerations do you think may have been over-emphasized?
   - What were your thoughts or feelings through genetic tests, ultrasounds, etc.?

   *Assume participant will share concerns of HCPs that they don’t have.*
3. Taking a look at the time immediately after childbirth, do you have any expectations or plans of what your life might be like and how you’ll respond to any changes?

   *Probes*- What changes are you expecting when it comes to your health and lifestyle?
   - Have you spoken to your healthcare provider, family or friends about this period of time?
   - What are some considerations and values you have for this period of time?

   *Assume participant will provide some considerations/outcomes around postpartum period relating to her health or that of neonate’s.*
4. How about down the line? Have you thought about anything that you feel is important for you or your child in the future? (This could be anything related to your health and well-being and lifestyle.)

   *Assume participant will provide some considerations/outcomes in the future, possibly relating to child’s health.*
5. Before we move on to the findings of our review of the literature, are there any other aspects of your health and care that we did not discuss that you want to share?

   *Assume either silence, nods no or if yes, an answer to this question.*
6. In the research, researchers and doctors have looked into: diet and exercise, wound complications, pain, blood pressure, GDM, gestational weight gain, postpartum weight retention, preeclampsia, initiation of breastfeeding, length of hospital stay, and more. Upon reflecting on one, any or all of these, what are your thoughts?

   *Probe*s
   - Have you discussed any or most of these with your health care provider or anyone else?
   - Which ones, and how often?
   - Is there anything missing from this list that you would like to add?

   *Assume either modifications to outcomes listed, or more outcomes.*
7. To sum up, could you provide us with two or three main considerations that you think are essential to consider, and should be measured in research? These can relate to you or your baby, during pregnancy and beyond.

   *Expect valued outcomes from participant.*
8. Is there anything else you would like to add?

   *Either silence, or some minor additions.*
9. Thank you for participating. We really appreciate your time.

   (*If telephoned or in person:* I have a feedback form asking about how this interview went as well as your possible participation in an online survey, a few months from now. Let any of us know if you have any questions.)

   (*If in person*, *give them form to fill out either in Rohan’s office or* outside) - We also have a little gift to thank you for your time, and some compensation for travel expenses – *give gift card and travel money in envelope*)

   (*If telephoned*: 1) Is there any feedback you feel comfortable sharing with me regarding this interview? 2) Would you be interested in an online survey a few months from now? If yes, what is your email address – if we don’t already have it?)
